# Gas in Military Mines

**Published:** 1916-05-20

**Authors:** 


### GAS IN MILITARY MINES.

### The Symptoms of Carbon Monoxide Poisoning.

A considerable medical literature sprang up in this country a year or so ago, after the introduction of poisonous gas by the Germans as a method of warfare. The symptoms, pathology, and treatment of this form of "gassing" were debated by some of those who had had large experience in casualty clearing stations and base hospitals; and a tolerable consensus of opinion about the results of and remedies for this novel method of irritant poisoning was arrived at. Another and quite distinct species of gas-poisoning is dealt with by Captain Sundell, R.A.M.C., in a recent paper* on the clinical manifestations of gas in military mines, based upon over one hundred cases of this condition which have passed through his hands within the last few months.

### The Problems of Military Mining.

He points out that military mining differs from industrial mining in three important particulars. The charges used are very much larger; the air space of the mine is very much smaller; and adequate ventilation of the shafts is much more difficult to maintain. Although a certain amount of irritant nitrous (or more likely nitric) fumes are present after an explosion, they are relatively unimportant compared with the carbon monoxide which is produced in very large quantities; most of the casualties from gassing are attributable to this latter gas—the "fire-damp" of our own coal-mines. If the explosion of the charge is incomplete, and part of the explosive burns instead of detonating, then the amount of carbon monoxide produced is much increased. In some parts of the trench line more casualties occur a few days after an explosion than actually at the time of it. This is noticed in chalky and friable soils, and is attributed to the penetration of the gas into fissures and crevices, whence it is gradually expelled by the subsequent settling of the soil or by driving new galleries. When all these perils of the military mine are taken into consideration, it is not to be wondered at that a high proportion of military crosses, distinguished conduct medals, and other greatly prized decorations in recent lists have been awarded to officers and men of the tunnelling companies to which these operations are confided. Civil mining is no child's play, but military mining must be a nerve-wracking business indeed.

The properties of "fire-damp" are well known: it is an odourless gas, highly inflammable; it is very fatal even if present in so small a proportion as one-tenth of 1 per cent. in the air that is breathed, and its action is both insidious and cumulative—insidious in that the victim does not notice any warning sensations as he enters air which is charged with the gas; and cumulative in that removal to a healthy atmosphere does not for a very considerable time restore the patient to the state he was in before he breathed the carbon monoxide. Incidentally, the well-known Davy lamp of the coal-miner gives warning of the presence of carbon monoxide; doubtless these lamps are used for the same purpose in France.

### Symptoms and Treatment.

Captain Sundell states that premonitory symptoms of this kind of "gassing" may be absent; but in many cases there is a feeling of heaviness and loss of power in the limbs, and sometimes of giddiness and lightheadedness. The loss of power may lead to disaster by preventing escape or by causing falls from the ladder when attempting to leave the mine.

All degrees of severity of cases are met with, from transient disability to complete collapse and death; the great majority, however, recover. The typical cherry-pink colour of the face and skin, described in all textbooks, has not been seen by this author; he describes his cases without exception as displaying pallor. So here again war experience is at variance with accepted civilian medical teaching. The patient presents a picture of extreme physical and mental exhaustion; the man crouches on a seat with his elbows on his knees and his head on his hands, with great disinclination to speak or move.

In severe cases the pulse rate rises to 140 or more, but in mild cases it is unaffected. The patients have a subnormal temperature; in bad cases severe rigors occur. Vomiting is an almost constant symptom, and often affords relief from the sensations of giddiness and weakness. Headache comes on after reaching the fresh air, and often lasts for several days. Palpitation and præcardial pain are occasionally complained of. Many of the patients completely "lose their nerve" after being gassed in this way.

### Treatment of Carbon Monoxide Inhalation.

Treatment is summed up in warmth, oxygen, artificial respiration, rest. Circulatory stimulants, such as hot coffee, are valuable. Strychnine has been found useful for stimulating the respiratory centre, but chief reliance is to be placed upon artificial respiration by Schäfer's method, combined with the administration of oxygen. Captain Sundell quotes one case of a man who was restored to life after two and three-quarter hours of apparently fruitless toil at artificial respiration. Rest is essential: even in mild cases it should be insisted upon for twenty-four hours, and for a good deal longer in severer forms. Men who have been slightly gassed should not be allowed to re-enter the mine until recovery is complete, on account of the cumulative effect of the poison. Phenacetin and aspirin for the subsequent headache should not be allowed, because their use has been known to be followed by great embarrassment of the heart's action.

* *Lancet*, May 6, 1916, p. 957.

